# S152. CANNABIDIOL INDUCED MODULATION OF MEDIOTEMPORAL ACTIVITY DURING A VERBAL MEMORY TASK IN FIRST-EPISODE PSYCHOSIS

**DOI:** 10.1093/schbul/sby018.939

**Published:** 2018-04-01

**Authors:** Aisling O’Neill, Robin Wilson, Grace Blest-Hopley, Luciano Annibale, Marco Colizzi, Sagnik Bhattacharyya

**Affiliations:** Institute of Psychiatry, Psychology & Neuroscience, King’s College London

## Abstract

**Background:**

Global neurocognitive impairments are a central feature of psychosis. Deficits in verbal memory in particular are the most consistently reported of these impairments from the first-episode of psychosis (FEP). Neuroimaging studies in psychosis have largely identified reductions in neural activation during various memory and learning related tasks, particularly in the medial temporal lobe, compared to healthy controls. Tetrahydrocannabinol (THC) and cannabidiol (CBD), both components of the cannabis plant that act through the endocannabinoid (eCB) system in the brain, have been found to induce direct and opposite neural effects during similar tasks in healthy samples, when compared to each other. Additionally, CBD has been shown to have antipsychotic properties, and may suppress THC induced psychotic symptoms and their directly associated functional abnormalities in healthy individuals. Thus far, the effects of CBD on the neural substrates implicated in memory and learning, and those underlying psychotic symptoms in FEP cohorts is unknown.

**Methods:**

17 FEP patients were initially recruited to the study. A double-blind, randomized, placebo controlled, repeated measures, within subject cross over design, with at least a one-week washout period between scans was employed. Participants were given identical capsules of either CBD (600mg), or placebo (PLB), then scanned using a block design fMRI paradigm, while performing a verbal paired associate learning task. 13 participants completed scanning, and were included in the analysis of the data. An ROI mask of the hippocampus, striatum, and parahippocampal gyrus was used in the data analysis, and all results were thresholded for less than one false positive over the whole map.

**Results:**

A CBD related decrease in activity was observed in the left hippocampus (p = 0.0024) and the right parahippocampal gyrus (p = 0.0024) during the recall condition, within the FEP group. No significant differences between PLB and CBD functional activity were observed during the encoding condition. No significant differences were observed between FEP participant performances on the CBD and PLB study days.

**Discussion:**

These findings provide robust evidence of the modulatory effect of an acute dose of CBD on the neural substrates underlying learning and memory, supporting a role for the eCB system in the abnormalities observed in psychosis, and its potential as a target for treatment.

